# Efficient and tumor-specific knockdown of MTDH gene attenuates paclitaxel resistance of breast cancer cells both in vivo and in vitro

**DOI:** 10.1186/s13058-018-1042-7

**Published:** 2018-09-18

**Authors:** Liu Yang, Yanhua Tian, Wei Sun Leong, Heng Song, Wei Yang, Meiqi Wang, Xinle Wang, Jing Kong, Baoen Shan, Zhengchuan Song

**Affiliations:** 1 Breast Center, Fourth Hospital of Hebei Medical University, Shijiazhuang, 050035 China; 2 Peking-Tsinghua Center for Life Sciences, Academy for Advanced Interdisciplinary Studies, Peking University, Beijing, 100871 China; 3 Department of Electrical Engineering and Computer Science, Massachusetts Institute of Technology, Cambridge, MA 02139 USA; 4 Laboratory of Experimental Pathology, Hebei Medical University, Shijiazhuang, China

**Keywords:** Breast cancer, Metadherin (MTDH) gene, Paclitaxel (TAX), Drug resistance, Nanoparticle (NP), Co-delivery

## Abstract

**Background:**

Drug resistance of paclitaxel (TAX), the first-line chemotherapy drug for breast cancer, was reported to develop in 90% of patients with breast cancer, especially metastatic breast cancer. Investigating the mechanism of TAX resistance of breast cancer cells and developing the strategy improving its therapeutic efficiency are crucial to breast cancer cure.

**Methods and Results:**

We here report an elegant nanoparticle (NP)-based technique that realizes efficient breast cancer treatment of TAX. Using lentiviral vector-mediated gene knockdown, we first demonstrated that TAX therapeutic efficiency was closely correlated with metadherin (MTDH) gene expression in breast cancer cell lines. This finding was also supported by efficacy of TAX treatment in breast cancer patients from our clinical studies. Specifically, TAX treatment became more effective when MTDH expression was decreased in MCF-7 cancer cells by the blocking nuclear factor-kappa B (NF-κB) pathway. Based on these findings, we subsequently synthesized a polymeric NP that could co-deliver MTDH-small interfering RNA (MTDH–siRNA) and TAX into the breast cancer tumors in tumor-bearing mice. The NPs were composed of a cationic copolymer, which wrapped TAX in the inside and adsorbed the negatively charged siRNA on their surface with high drug-loading efficiency and good stability.

**Conclusions:**

NP-based co-delivery approach can effectively knock down the MTDH gene both in vitro and in vivo, which dramatically inhibits breast tumor growth, achieving effective TAX chemotherapy treatment without overt side effects. This study provides a potential therapeutic strategy for the treatment of a wide range of solid tumors highly expressing MTDH.

**Electronic supplementary material:**

The online version of this article (10.1186/s13058-018-1042-7) contains supplementary material, which is available to authorized users.

## Background

The key options in breast cancer treatment so far include surgery, chemotherapy, radiotherapy, and molecular targeted therapy [1]. Out of these approaches, chemotherapy appears to be the most widely adopted. In particular, paclitaxel (TAX) was commonly used as one of the first-line chemotherapy drugs in breast cancer therapy and has shown remarkable efficacy in inhibiting tumor growth through mitotic arrest [2–4]. Despite the excellent initial therapeutic efficacy, drug resistance of TAX develops in 90% of breast cancer patients during the disease progression [5]. The molecular mechanism of such resistance remains elusive and has become a clinical issue that requires an immediate solution.

Metadherin (MTDH), also known as LYRIC, AEG-1, or 3D3, has been expressed in multiple tumor types as an oncogene and associated with aberrant proliferation and drug resistance of tumor cells [6–10]. In particular, a recent study reported that MTDH gene promotes the cisplatin resistance of cervical cancer cells by activating the Erk/nuclear factor-kappa B (Erk/NF-κB) pathway and decreasing cleavage of caspase-3 [11–14]. Based on these findings, we explored the relationship between MTDH gene expression and TAX resistance in breast cancer cell lines as well as the effect of MTDH knockdown on TAX therapeutic efficiency. For in vivo therapeutic study, we employed targeted nanocarriers to specifically co-deliver anti-MTDH small interfering RNA (siRNA) (for tumor-specific silence of MTDH gene) and TAX drug.

We first explored the role of MTDH gene in the TAX therapeutic efficiency toward breast cancer cells and then investigated whether a nanoparticle (NP)-based co-delivery method can be used to address TAX resistance issue in breast cancer treatment. We hypothesized that the two drugs-loaded NPs displayed onsite gene silencing in tumor tissues, which improved drug sensitivity of TAX with good tolerability. In addition, poly(lactic-co-glycolic acid) (PLGA) polymeric molecule is approved by the US Food and Drug Administration with high biosafety; this study thus presents a promising strategy for clinical practice.

## Methods

### Patients and clinicopathology characteristics

This is a retrospective study that was approved by the Fourth Hospital of Hebei Medical University in Shijiazhuang, China. In this study, we selected 44 cases with breast cancer, which were proven to be breast invasive ductal carcinoma by pathological diagnosis from March to December 2010. The median follow-up time was 84 months (range of 8–90 months). The primary endpoints were disease-free survival (DFS) and overall survival (OS). We detected the samples from carcinoma of 44 patients before neoadjuvant chemotherapy. Immunohistochemistry was used to detect MTDH expression in all tissues. All the patients were recommended to use combination chemotherapy of taxol and anthracycline. Clinical benefit was evaluated by the Response Evaluation Criteria in Solid Tumors (RECIST version 1.1) and pathologic complete response (pCR) rates after neoadjuvant therapy. pCR was defined as ypT0 ypN0. All of above procedures were approved by the Fourth Hospital Ethics Committee of Hebei Medical University in Shijiazhuang, China (SCXK2009–0037).

### Immunohistochemistry

All of the immunohistochemistry slides for MTDH were reviewed again by two independent pathologists. Immunohistochemistry staining of 4-μm sections of formalin-fixed paraffin-embedded tissue was rehydrated and incubated with anti-MTDH primary monoclonal antibodies (Cell Signaling Technology, Danvers, MA, USA) or phosphate-buffered saline (PBS) at 4 °C overnight, followed by sequential incubation with MaxVision™/horseradish peroxidase (HRP) and diaminobenzidine (DAB). Then slides were counterstained with hematoxylin, dehydrated, and mounted.

The levels of MTDH expression were evaluated on the basis of the staining intensity (SI) and percentage of positively stained tumor cells (PP). SI was defined as 0 (no staining), 1 (weak staining), 2 (moderate staining), and 3 (strong staining). PP was graded according to the following criteria: 0 (no positive tumor cells), 1 (1%–20% positive tumor cells), 2 (21%–50% positive tumor cells), 3 (51%–70% positive tumor cells), and 4 (>70% positive tumor cells). The immunoreactive score (IRS) was calculated as follows: IRS = SI × PP. IRS of 0 means negative expression, IRS of 1–3 means weakly positive, IRS of 4–6 means moderately positive, and IRS of 8–12 means strong positive. Here, low expression was defined as an IRS of 3 or less. High expression was defined as an IRS of 4 and more [15].

### Cell lines and reagents

The human breast cancer cell lines MCF-7 and MDA-MB-435S were purchased from the American Type Culture Collection (Manassas, VA, USA) and propagated in RPMI-1640 medium (Thermo Fisher Scientific, Waltham, MA, USA) supplemented with 10% fetal bovine serum (Biological Industries Israel Beit-Haemek Ltd., Kibbutz Beit-Haemek, Israel) and antibiotics. All cells were maintained in 5% CO_2_ at 37 °C. TAX was purchased from Yangzijiang Medicine Co. Ltd. (Jiangsu, China).

### Lentiviral-mediated overexpression or silencing of MTDH gene in MCF-7 cells

The plasmids containing MTDH gene or MTDH-short hairpin RNA (MTDH-shRNA) (5′-gcaattgggtagacgaagaaa-3′) were designed and amplified by transfecting into *Escherichia coli*. DH5α. Real-time polymerase chain reaction (RT-PCR) and Western blot were used to detect the expression of MTDH mRNA and protein of MTDH-shRNA to verify the effect of transfection. Plasmids enveloped in lentivirus were incubated with MCF-7 cells for 6 h according to the MOI (multiplicity of infection) value and the virus titer and subsequently placed in fresh medium. Puromycin (0.4 μg/mL) was used to screen stable transfection cell lines.

### RT-PCR analysis

Total RNA from treated cells was extracted with Trizol (Takara, Dalian, China) in accordance with the instructions of the manufacturer. Total RNA was used to synthesize cDNA by using PrimeScript RT reagent Kit (Takara). Then RT-PCR was carried out using Power Up SYBR Green Master Mix (Life Technologies, Thermo Fisher Scientific). The reaction was conducted using the following parameters: 95 °C for 30 s, 95 °C for 5 s, and 60 °C for 30 s during 40 cycles. Internal control and primers for RT-PCR were obtained from the reference. RT-PCR was then employed to determine the change of MTDH mRNA in MCF-7–MTDH cell line and MCF-7–MTDH–shRNA cell line. The experiments were repeated for three times and data were analyzed using 2^−∆∆Ct^. The data were normalized to the geometric mean of housekeeping gene *β-actin* to control the variability in expression levels. RT-PCR primers were synthesized by SBS Genentech Co. Ltd. (Shanghai, China). The specific primers for MTDH and reference gene (β-actin) are as follows:

- MTDH forward: 5′-AAATAGCCAGCCTATCAAGACTC-3′;
- MTDH reverse: 5′-TTCAGACTTGGTCTGTGAAGGAG-3′.
- β-actin forward, 5′-GCTACAGCTTCACCACCACAG-3′;
- β-actin reverse, 5′-GGTCTTTACGGATGTCAACGTC-3′.

### Western blot analysis

Cells were lysed and total proteins were separated by 10% SDS-PAGE and transferred (300 mA, 2 h) onto a PVDF membrane. After blotting with 5% nonfat milk, the membranes were incubated with primary antibodies (anti-MTDH 1:20000, anti-p65 1:5000, anti-p-p65 (S536) 1:1000, anti-IκBα1:1000, and β-actin 1:1000) at 4 °C overnight. Then the membranes were washed by TBS-T buffer and incubated with secondary HRP-labeled anti-rabbit antibody at room temperature for 1 h and washed with TBS-T buffer three times (10 min each time). The target proteins were visualized with a chemiluminescence system (Gene Company Ltd., Shanghai, China) and normalized to β-actin from the same membrane.

### Cell apoptosis and cycle detection

Cell apoptosis was performed using an Annexin V-PE/7-AAD Apoptosis Detection Kit (KeyGEN BioTECH, Nanjing, China). The experiments were carried out strictly in accordance with the instructions of the manufacturer. The cells were then analyzed by Beckman Coulter Cytomics FC 500 flow cytometry (Beckman Coulter, Inc., Brea, CA, USA). The data were analyzed by EXPO32 ADC analysis software. Cell cycle analysis was performed by using the standard method with some modifications. In brief, cells were fixed with 75% ethanol at 4 °C overnight. The fixed cells were washed by PBS and suspended with 200 μL RNaseA at 37 °C for 10 min, and 250 μL PI (100 μg/mL) was added to stain the DNA of cells in the dark for 15 min. Cell cycle was analyzed with a Beckman Coulter Cytomics FC 500 flow cytometry, and the data were analyzed by Multicycle AV for Windows (version 295) software.

### Cell viability assay

Cell viability was determined by a Cell Counting Kit-8 (CCK-8) assay. MCF-7, MCF-7-vector, MCF-7–MTDH, and MCF-7–MTDH–shRNA cells were seeded into 96-well plates at a density of 1 × 10^4^/well (TAX 0 μg/mL) or 5 × 10^4^/well (TAX 1 μg/mL) in 100 μL RPMI-1640 medium. After incubation in 5% CO_2_ at 37 °C overnight, the RPMI-1640 medium in each well was replaced with a different concentration of TAX (0 and 1 μg/mL) and further incubated for 0, 24, 48, and 72 h. Afterwards, 10 μL of CCK-8 was added to each well for another 2 h at 37 °C. The absorbance at 450 nm was read by the Microliate Reader (BioTek, Winooski, VT, USA). The inhibitory rate of cell growth was calculated on the basis of the following equation: cell growth inhibition rate = (1 − experimental OD450 / control OD450) × 100%. The experiments were repeated three times.

### Tumorigenicity assay

MCF-7, MCF-7-vector, MCF-7–MTDH, and MCF-7–MTDH–shRNA cells (5 × 10^6^ in 0.1 mL) were injected subcutaneously into 4-week-old female nude mice, respectively. TAX treatment was started at the third week after cell injection. The mice were randomly assigned to an untreated group and TAX treatment groups. The dose of TAX was 10 mg/kg and administered by intraperitoneal (IP) injection once a week for a total of four injections. Tumor volume was measured every three days (volume = 0.5 × length × width^2^, measured with a Vernier caliper). After the last treatment, the mice were sacrificed and the tumors were removed for weight analysis.

### Preparation of NPs

For poly(etherimide)-poly(lactic-co-glycolic acid) (PEI-PLGA) NP synthesis, 20 mg was dissolved in 1 mL of methylene chloride and mixed with 0.2 mL deionized water. The mixture was emulsified through sonication by using probe sonicator at 25% power for 5 min. Then 2 mL of 2% poly(vinyl alcohol) (PVA) and 0.2 mL of hydrophobic TAX (with different ratios) dissolved in methylene chloride were added into the mixture. The solution was emulsified again at 30% power for 5 min, added dropwise into 10 mL of 0.6% PVA, and stirred for 3 min. The organic solvent was removed in a rotary evaporator under reduced pressure. The core particles containing TAX were centrifuged at 12,000 revolutions per minute (rpm) for 5 min and rinsed with deionized water. For loading siRNA, different ratios of TAX were added into the solution of core NPs and stirred at a rate of 200 rpm for 20 min. The core NPs absorbed by siRNA on their surface were centrifuged at 12,000 rpm for 5 min.

### Morphological characterization of NPs

The NPs were negatively stained with uranyl acetate solution (2%) and deposited on a carbon-coated copper grid. The morphology was characterized with a transmission electron microscope (TEM) (JEM-200CX; Jeol Ltd., Tokyo, Japan). Size distribution (diameter in nanometers) and surface charge (zeta potential in millivolts) of NPs were determined by using a ZetaSizer Nano series Nano-ZS (Malvern Instruments Ltd., Malvern, UK) equipped with a He-Ne Laser beam at a wavelength of 633 nm and a fixed scattering angle of 90. Determinations were performed at 25 °C for samples appropriately diluted in distilled water.

### Tumor-bearing nude mouse model

Six-week-old female BALB/c nude mice were purchased from Beijing Vital River Laboratories (Beijing, China). Human breast cancer cells (MCF-7, 2.0 × 10^6^ cells in 50 mL PBS) mixed with 50 mL of Matrigel were transplanted into the mammary fat pads of the mice and allowed to grow to a tumor size of about 100 mm^3^ (volume = 0.5 × length × width^2^, measured with a Vernier caliper). The mice were then randomly divided into different experimental groups. All procedures were approved by the Committee on the Ethics of Animal Experiments of the Health Science Center of Peking University (Beijing, China).

### Statistical analysis

Data analyses were performed with one-way analysis of variance (ANOVA) and the least significance difference (LSD) multiple comparisons test with PASW Statistics 23. Tumor volumes were compared by using a Kruskal–Wallis test followed by the Mann–Whitney test.

## Results

### Association of MTDH expression with the probability of disease-free survival and overall survival and efficacy of TAX treatment in patients with breast cancer

**Fig. 1:**
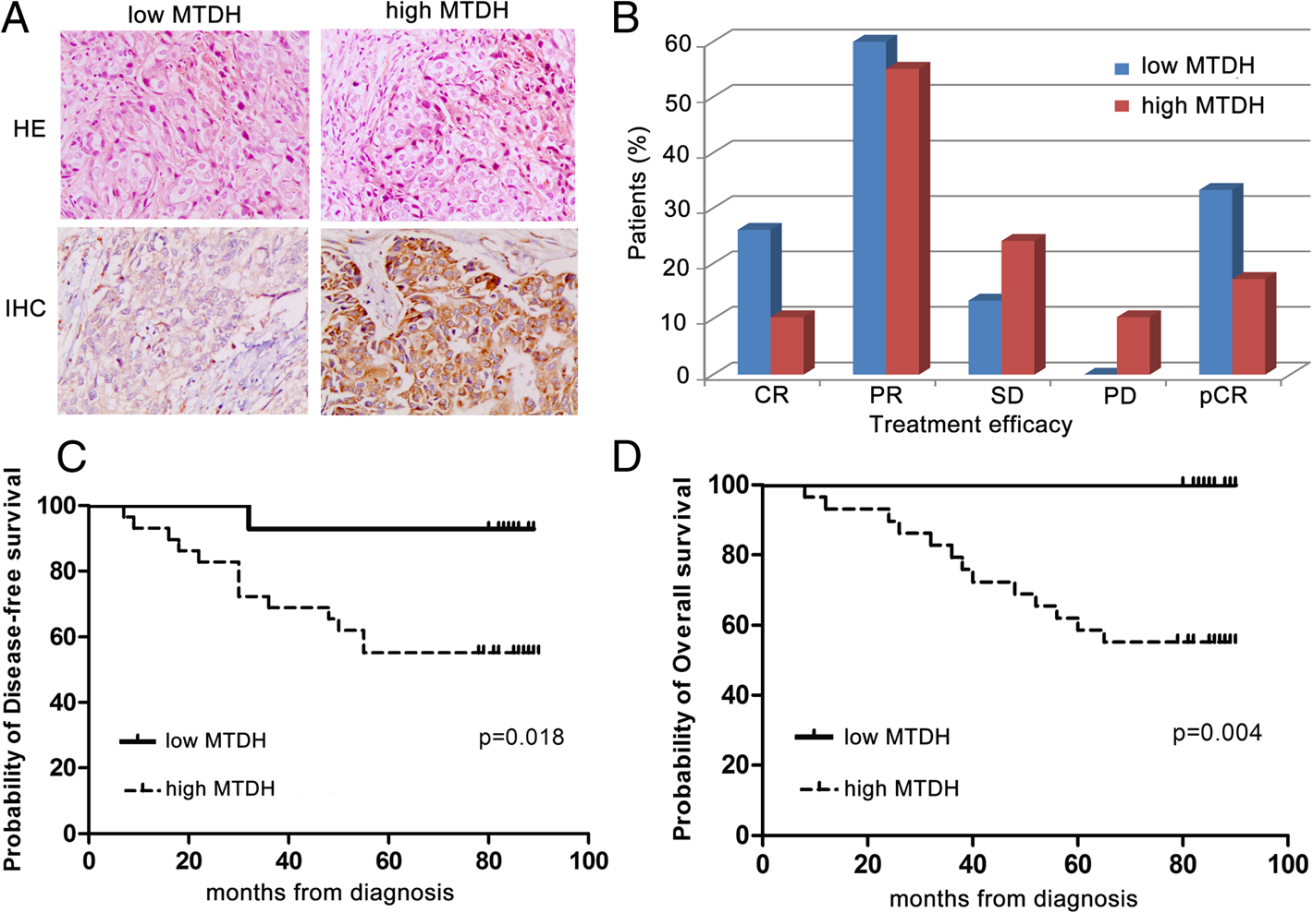
Metadherin (MTDH) overexpression affects the prognosis of breast cancer patients who were treated with neoadjuvant chemotherapy. **a** Immunohistochemical staining with MTDH expression in breast cancer tissues (200×). Abbreviations: *HE* histopathology, *IHC* immunohistochemistry. **b** After neoadjuvant chemotherapy, in the MTDH low group (*n* = 15), 4 cases achieved complete response (CR), 9 cases achieved partial response (PR), 2 cases achieved stable disease (SD), and 0 case achieved progression disease (PD). In the high MTDH expression group (*n* = 29), 3 cases achieved CR, 16 cases achieved PR, 7 cases achieved SD, and 3 cases achieved PD. After surgery, the percentages of pathologic complete response (pCR) were 33.3% in the low MTDH expression group and 17.2% in the high MTDH expression group. **c** and **d** Patients with high MTDH expression had worse disease-free survival (*P* = 0.018) and overall survival (*P* = 0.004) than those with low MTDH expression

In a cohort of 44 neoadjuvant chemotherapy breast cancer patients, 29 patients were MTDH gene positive and 15 patients were negative. Patients with high MTDH protein expression (tan or brown staining in the cell membrane or cytoplasm, Fig. 1a) had significantly worse probability of disease-free survival (DFS) and overall survival (OS) than those with low MTDH protein expression (Fig. 1c, d). Furthermore, we found that MTDH protein expression negatively correlated with the TAX-containing chemotherapy efficacy (Fig. 1b). Based on these MTDH gene-associated clinical characteristics, we conducted the subsequent study to confirm the function of MTDH gene in breast cancer and its possible relationship with TAX chemotherapy.

### Effect of MTDH expression on MCF-7 breast cancer cells

**Fig. 2:**
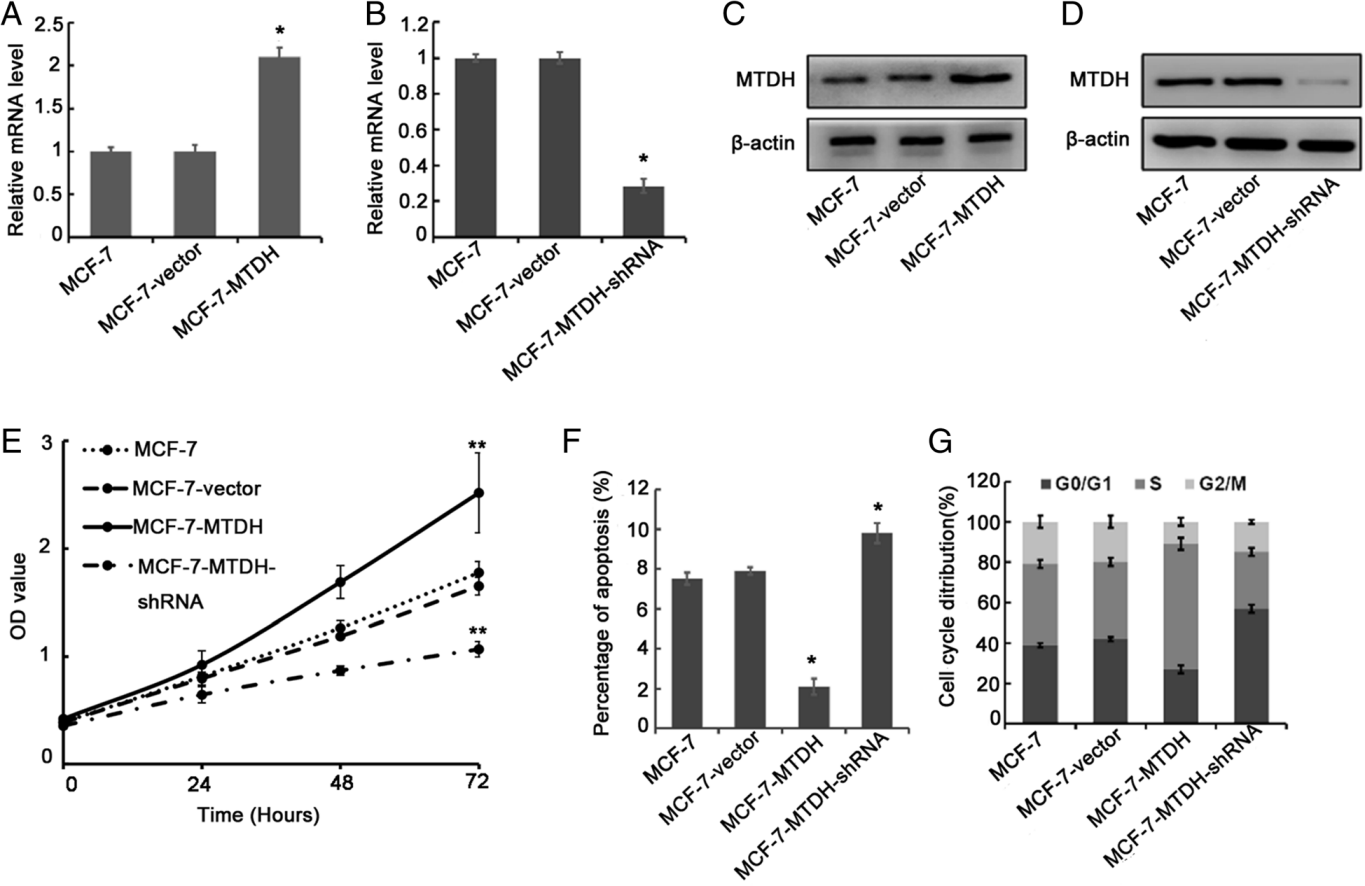
Effect of metadherin (MTDH) expression in MCF-7 breast cancer cells. **a** and **c** Relative MTDH mRNA and protein expression levels after transfection of MCF-7 cells with MTDH plasmid. **b** and **d** After transfection with MTDH-short hairpin RNA (MTDH-shRNA) plasmids, the relative MTDH mRNA and protein expression levels were significantly lower than control cells. **e** Cell growth rate was evaluated by using a Cell Counting Kit-8 assay. Knockdown of MTDH inhibited MCF-7 cell proliferation, while MTDH overexpression induced cell growth. Abbreviation: *OD* optical density. **f** Flow cytometric analysis of apoptosis of the MCF-7–MTDH and MCF-7–MTDH–shRNA cells. MTDH overexpression decreased cell apoptosis, while knockdown of MTDH did the opposite. **g** Overexpression of MTDH arrested cells in S phase, while MTDH silencing increased the proportion of cells in G_0_/G_1_ phase. In all of these tests, the MCF-7 and MCF-7-vector cells were used as a reference sample. *P <0.05 and **P <0.01 compared with MCF-7

To investigate the effect of MTDH expression in breast cancer cell lines, we first performed RT-PCR tests on our modified MCF-7 breast cancer cells. We constructed four groups of shRNA and one control shRNA. After transfecting, amplifying, and extracting the four groups of plasmids, we selected the optimal silent shRNA (MTDH-shRNA3: 5′-GCAATTGGGTAGACGAAGAAA-3′) via RT-PCR and Western blot (Additional file 1: Figure S1). As shown in Fig. 2a, the relative MTDH mRNA expression level in our MCF-7–MTDH cell was 2.1 times higher than the reference MCF-7 cell. On the other hand, the relative MTDH mRNA expression level in our MCF-7–MTDH–shRNA cell was only 0.3 times the MCF-7 cell (Fig. 2b). Western blot tests were then conducted on MCF-7 cell and modified MCF-7 cells. We found that the protein level in MCF-7–MTDH cell was 2.86 times higher than the reference MCF-7 cell but in MCF-7–MTDH–shRNA cell was 90% lower than the reference cell (Fig. 2c, d). Furthermore, through CCK-8 assay, we observed that the growth rates of MCF-7–MTDH and MCF-7–MTDH–shRNA cells were higher and lower than that of the reference cell, respectively (Fig. 2e). Apart from that, through the cell apoptosis test, a test that measures the programmed cell death rate, we showed that the apoptosis percentage in an MCF-7–MTDH cell was much lower than MCF-7 cell but that of MCF-7–MTDH–shRNA was higher compared with MCF-7 cell (Fig. 2f, Additional file 1: Figure S2). In Fig. 2g and Additional file 1: Figure S4, we compared the proportion of G_0_/G_1_, S and G_2_/M phases in MCF-7–MTDH and MCF-7–MTDH–shRNA with the reference MCF-7 cell, respectively. Compared with MCF-7 cell, the MCF-7–MTDH cell had more S phase and less G_0_/G_1_ and G_2_/M phases while the MCF-7–MTDH–shRNA cell contained much lesser S phase and more G_0_/G_1_ and G_2_/M phases.

### Relationship between MTDH expression and TAX treatment in MCF-7 cell

**Fig. 3:**
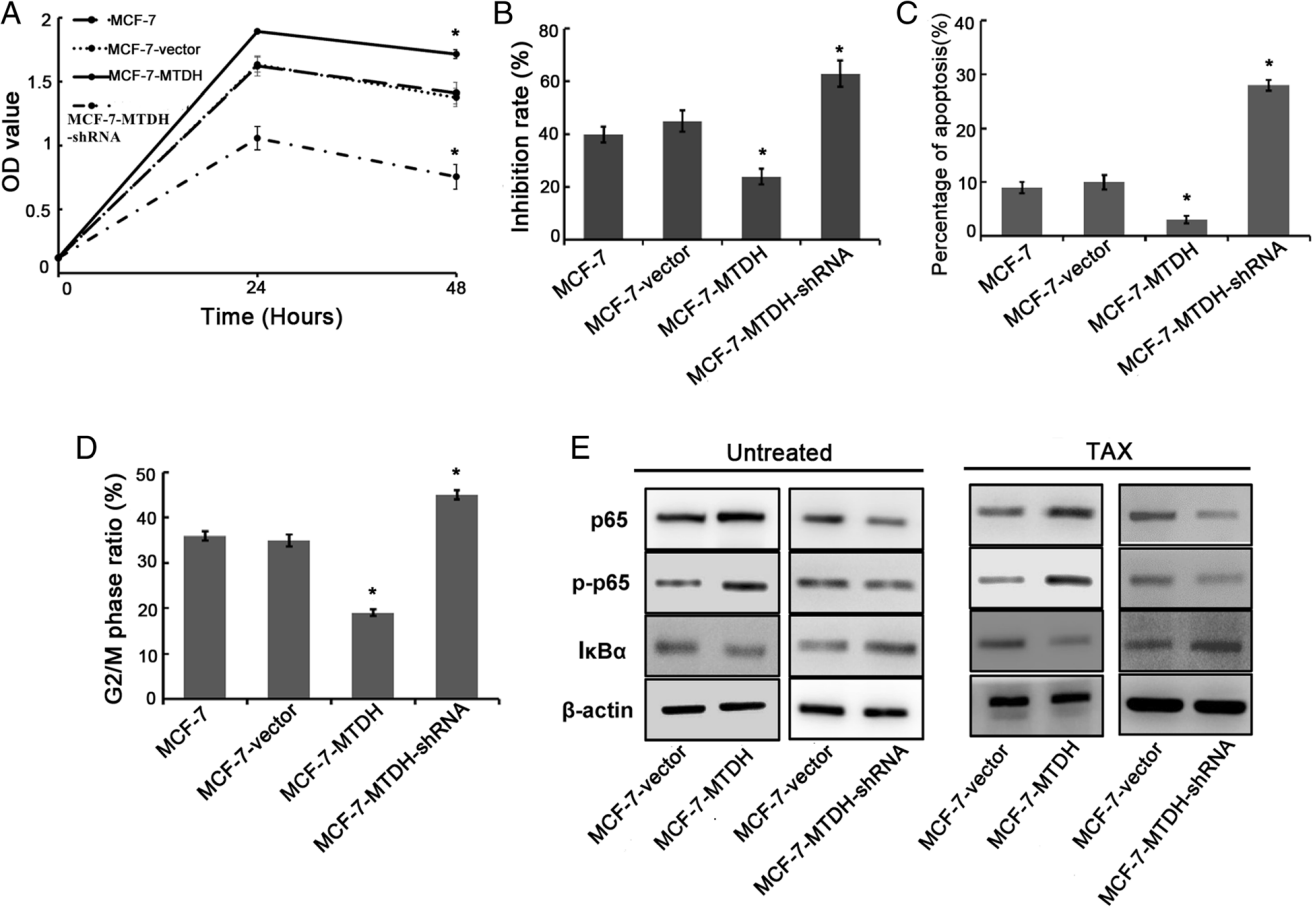
Effect of metadherin (MTDH) expression on the efficacy of paclitaxel (TAX) in MCF-7 cells. **a** Cytotoxicity was evaluated by Cell Counting Kit-8 assay after treatment with TAX for 48 h. Abbreviation: *OD* optical density. **b** The inhibition rates of MCF-7–MTDH–short hairpin RNA (MCF-7–MTDH–shRNA) cells were much higher than those of MCF-7–MTDH cells. **c** MTDH suppressed cell apoptosis induced by TAX, while MTDH silencing increased the sensitivity of TAX. **d** Knockdown of MTDH increased the G_2_/M phase arrest induced by TAX, while the opposite effect was found in MTDH overexpressing MCF-7 cells. **e** Western blot analysis revealed that protein expression of p65 was higher in MCF-7–MTDH cells, while IκBα was lower. On the contrary, in MCF-7–MTDH–shRNA cells, the protein expression level of p65 decreased and IκBα increased. Knockdown of MTDH could inhibit the activity of the nuclear factor-kappa B (NF-κB) pathway. *P <0.05 and **P <0.01 compared with MCF-7

We now study how the MTDH expression in MCF-7 cell affects the effectiveness of the TAX drug treatment on breast cancer. We evaluated and compared the cytotoxicity of the modified MCF-7 cell with a reference MCF-7 cell through a CCK-8 assay. In Fig. 3a and b, we can see that the inhibition rate of the MCF-7–MTDH cell (25.89 ± 1.33%) was much lower than that of the MCF-7 cell (40.46 ± 1.31%) while that of MCF-7–MTDH–shRNA cell was the highest (64.33 ± 2.21%) at 48 h. In addition, the apoptosis percentage in MCF-7–MTDH cell (2.91 ± 0.89%) was lower than MCF-7 cell (9.31 ± 1.04%) while that of MCF-7–MTDH–shRNA cell (27.56 ± 2.40%) was higher compared with MCF-7 cell after TAX treatment (Fig. 3c, Additional file 1: Figure S3). As expected, the proportion of G_2_/M phase ratio in the MCF-7–MTDH cell was significantly lower than the MCF-7 reference cell after TAX treatment (Fig. 3d, Additional file 1: Figure S5). The NF-κB pathway was closely associated with chemotherapy resistance, so we examined the expression level of p65, p-p65, and IκBα. As shown in Fig. 3e, upregulation of MTDH increased p65 and p-p65 expression but reduced the expression of IκBα (suggesting the activation of NF-κB pathway). On the contrary, silencing of MTDH reduced p65 and p-p65 and increased IκBα expression. These results suggested that MTDH was related to NF-κB and TAX sensitivity.

### Overexpression of MTDH promotes MCF-7 tumor growth in vivo and diminishes TAX activity

**Fig. 4:**
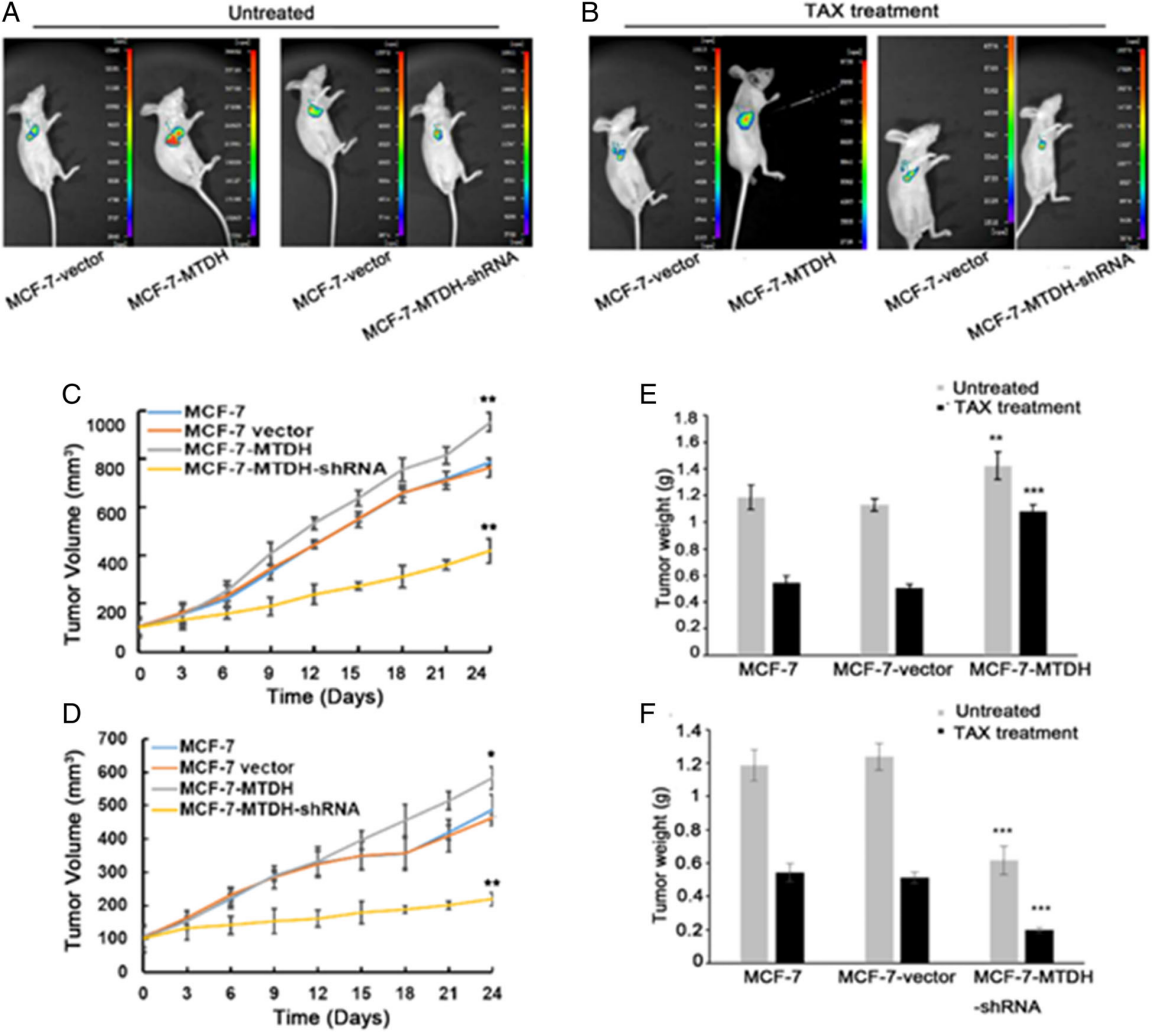
Effect of metadherin (MTDH) overexpression on MCF-7 tumorigenicity and paclitaxel (TAX) resistance. **a** and **b** *In vivo* imaging system was used to analyze the growth of xenograft tumors in the untreated group (a) and the TAX treatment group (b). **c** Without TAX treatment, the tumor volume was larger in the MCF-7–MTDH group compared with the MCF-7 control group. **d** In the TAX-treated group, the tumor volumes of the MCF-7–MTDH-short hairpin RNA (MCF-7–MTDH–shRNA) group were significantly smaller than those of the control group. **e** and **f** The tumor weights in both MCF-7–MTDH and MCF-7–MTDH–shRNA show consistent trends with tumor volume. *P <0.05, **P <0.01, and ***P <0.001 compared with MCF-7

We further examined the effect of MTDH expression on in vivo MCF-7 tumor cell growth and TAX treatment efficiency using a mouse xenograft model. Figure 4a and b compared the in vivo images of xenograft tumors in untreated mice (MCF-7) and TAX-treated mice (MCF-7–MTDH and MCF-7–MTDH–shRNA). As can be seen, the tumor was significantly larger in the MCF-7–MTDH group but was smaller in the MCF-7–MTDH–shRNA group after subcutaneous injection of cells for 14 days. Mice were then treated with TAX by IP injection once a week for a total of four injections. The tumor in the MCF-7–MTDH–shRNA group was dramatically smaller than MCF-7–MTDH group as well as both controls (Fig. 4b), confirming that the knockdown of MTDH enhanced cell sensitivity to TAX exposure. Tumor volume measurement results also supported these observations (Fig. 4c, d). In addition, the tumor weight in the MCF-7–MTDH–shRNA group with or without TAX treatment was lower than that of the MCF-7–MTDH group or control group (Fig. 4e, f).

### Development of MTDH–siRNA and TAX co-delivery technique

**Fig. 5:**
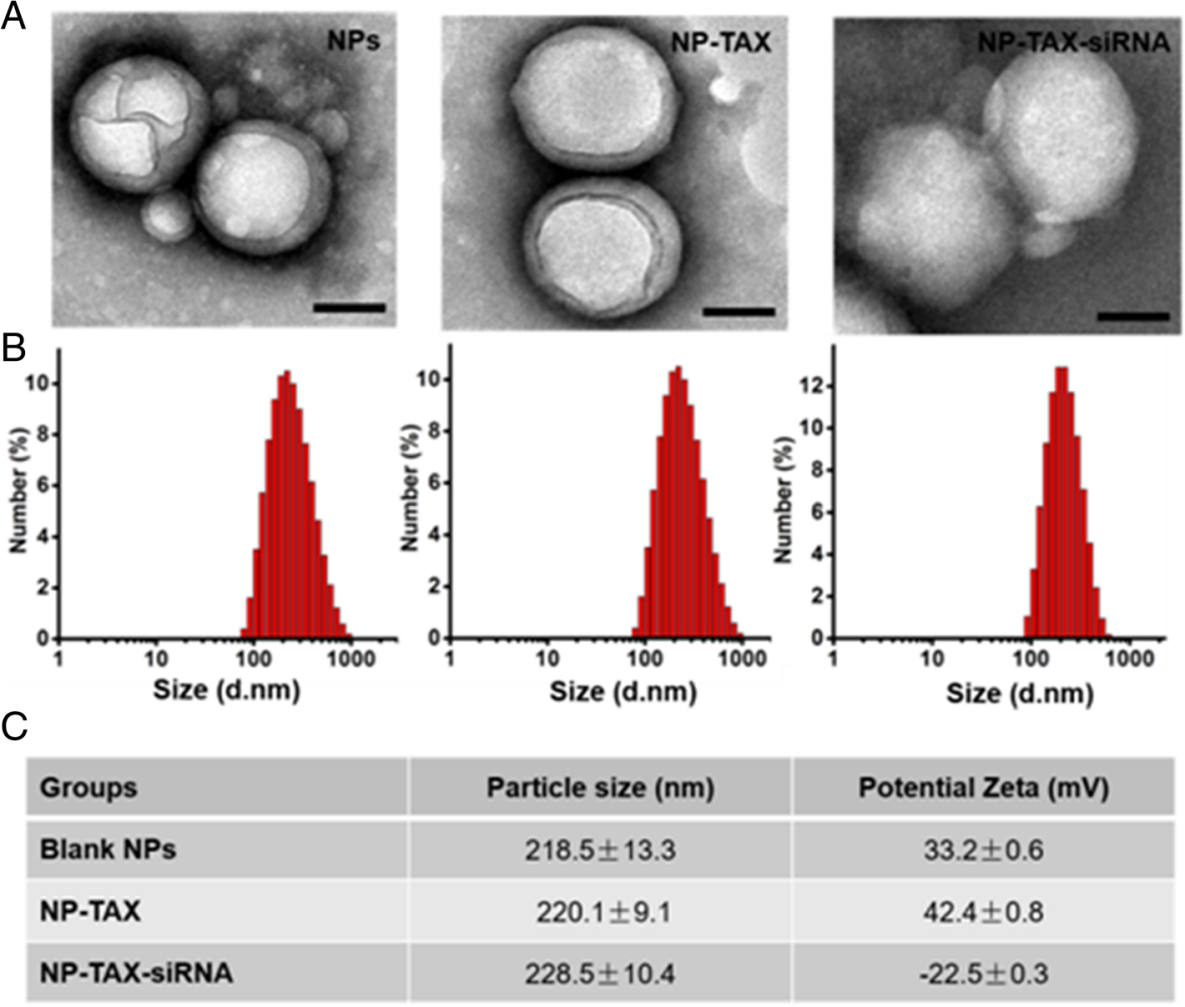
Morphology, size, and surface charge characterization of drug-loaded nanoparticles (NPs). **a** Blank NPs (NPs), NP-TAX, and NP-TAX–siRNA. Scale bars = 100 nm. **b** Size distribution of blank NPs, NP-TAX, and NP-TAX–siRNA. **c** Particle size and potential zeta of NPs

Based on the findings above, we have developed a new technique (amphiphilic copolymer PEI-PLGA) that allows both MTDH–siRNA and TAX to be concurrently delivered into breast cancer cells to effectively control the breast cancer condition. After emulsification twice, the TAX was loaded into the hydrophobic layer and the MTDH–siRNA was bound at the NP surface after addition through electrostatic interactions. Figure 5 shows the TEM images of blank NPs, TAX-encapsulated NPs (NP-TAX), and NP-TAX–siRNA. As can be seen, all NPs were dispersed with a well-defined spherical core shell structure. Dynamic light scattering (DLS) measurement suggested that the average hydrodynamic diameters of blank NPs, NP-TAX, and NP-TAX–siRNA were 218.5 ± 13.3 nm, 220.1 ± 9.1 nm, and 228.5 ± 10.4 nm, respectively (Fig. 5b, c). The zeta potentials of the blank NPs, NP-TAX, and NP-TAX–siRNA were 33.2 ± 0.6 mV, 42.4 ± 0.8 mV, and −22.5 ± 0.3 mV, respectively (Fig. 5c). When siRNA was mixed with NP-TAX, the zeta potential of NP-TAX–siRNA changed from 42.4 to −22.5 mV, indicating the successful and a large capacity of absorption of negatively charged nucleic acids on the NP surface. We also investigated the release profile of TAX at different pHs over time (Additional file 1: Figure S6). At pH 7.4, no significant release of TAX was observed in the first 10 h; however, TAX was released at a fast rate at pH 4.4, and a release ratio of around 40% was reached in the first 10 h. Therefore, the polymeric core is expected to show a pH-dependent drug release, which facilitates complete drug release in lysosomes after cellular uptake.

### Cellular uptake and gene silencing of NP-TAX–siRNA

**Fig. 6:**
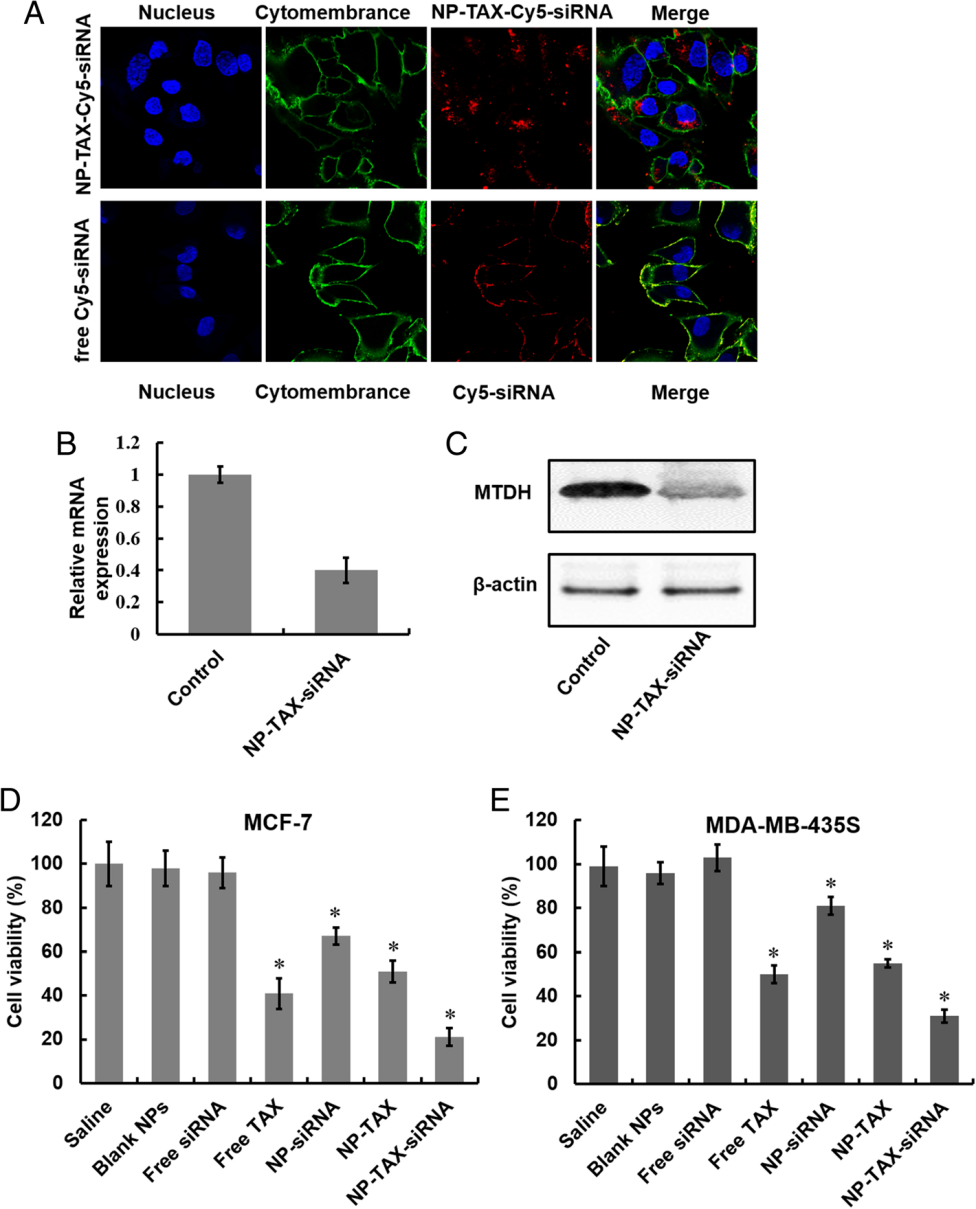
Cellular uptake, intracellular distribution, and cytotoxicity of nanoparticle-paclitaxel–small interfering RNA (NP-TAX–siRNA). **a** Cellular uptake of NP-TAX–siRNA. Confocal microscopic images of MCF-7 cells treated with NP-TAX–siRNA for 6 h. Cell nucleuses (blue) were stained by DAPI, cytomembrane was labeled with DiO green fluorescence, and siRNA was labeled with Cy5 red fluorescence. **b** and **c** Gene silencing ability of NP-TAX–siRNA. MCF-7 cells were treated with free metadherin (MTDH)-siRNA and NP-TAX–siRNA in serum-free media for 6 h. After 48 h, the mRNA levels of MTDH were measured by real-time polymerase chain reaction (RT-PCR) (**b**). The expression of MTDH protein was analyzed by Western blot (**c**). **d** and **e** Cell viability of MCF-7 (**d**) and MDA-MB-435S (**e**) breast cancer cells was measured by using Cell Counting Kit (CCK) assay. Cells were incubated with different drug formulations (Saline, Blank NPs, Free siRNA, Free TAX, NP-siRNA, NP-TAX, and NP-TAX–siRNA) at 37 °C for 48 h. The cell viability of saline was set as 100%. Each bar represents the mean ± standard deviation of three replicates. *P <0.05

Two prerequisites for efficient siRNA-mediated gene silencing effect are high siRNA uptake levels and successful release of siRNA to cytoplasm. To study cellular uptake of NPs, we labeled NP-TAX–siRNA with near-infrared fluorescent dye Cy5. After incubation of MCF-7 cells with NP-TAX–Cy5–siRNA for 6 h, obvious red fluorescence appeared in the cytoplasm (Fig. 6a, upper panel). In contrast, there was barely red fluorescence in cells treated with free Cy5–siRNA (Fig. 6a, lower panel) and this was probably due to their high molecular weight, hydrophilic nature, and high density of charge. The knockdown efficiency of MTDH–siRNA encapsulated in NPs was then tested in MCF-7 cells by using RT-PCR and Western blot analysis. The downregulation of MTDH mRNA and protein expression in cells was observed after the NP-TAX–siRNA treatment, indicating the successful release of siRNA from lysosomes to cytoplasm (Fig. 6b, c). In addition, we examined the cytotoxicity of NP-TAX–siRNA to breast cancer cells in vitro. The MCF-7 cells were incubated with saline, blank NPs, free TAX, free siRNA, NP-TAX, NP-siRNA, or NP-TAX–siRNA for 48 h, followed by quantification of cell viability using a CCK-8 cell proliferation assay. As shown in Fig. 6d, compared with the saline group, free siRNA did not show significant inhibition in tumor cell growth. A possible reason for this phenomenon is that free siRNA cannot be taken up by cells easily. In contrast, the siRNA encapsulated in NPs (NP-siRNA) exhibits effective inhibition of cell growth, indicating successful delivery of siRNA into cells. Free TAX was more toxic than TAX encapsulated in NPs, while the inhibitory effect of NP-TAX–siRNA on cell growth outperformed all other groups. In addition, no cytotoxicity was observed in the blank NP-treated group, suggesting that the polymers are non-toxic. To confirm the inhibition effect of NP-TAX–siRNA on cell growth, we also tested cell proliferation after NP-TAX–siRNA treatment by using another human breast tumor cell line MDA-MB-435S (Fig. 6e), which has been demonstrated to express MTDH. Comparable results were observed.

### In vivo biodistribution and antitumor activity of NP-TAX–siRNA

**Fig. 7:**
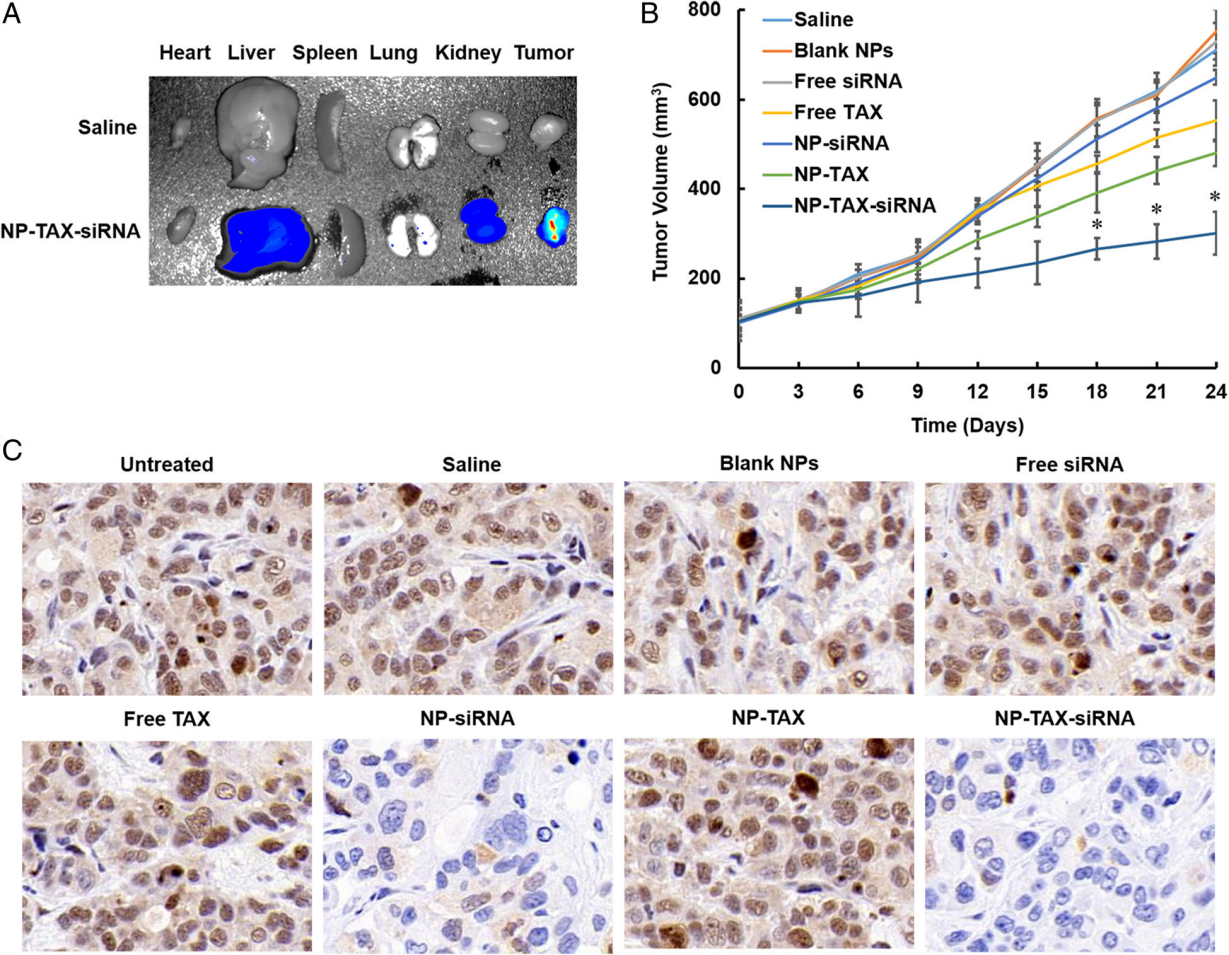
Biodistribution and antitumor activity of nanoparticle-paclitaxel–small interfering RNA (NP-TAX–siRNA). **a** *Ex vivo* fluorescence imaging of major organs from MCF-7 tumor-bearing mice 10 h after intravenous injection with saline or Cy5.5-labeled NP-TAX–siRNA. **b** Mice bearing tumors were injected intravenously with different reagents (PBS, Blank NPs, Free siRNA, Free TAX, SP-siRNA, NP-TAX, and NP-TAX–siRNA) every other day from day 3 for a total of six injections. NP-TAX–siRNA treatment dramatically inhibited tumor growth compared with the control groups. Statistical analyses were performed by using a Kruskal–Wallis test followed by the Mann–Whitney test. *P <0.05 (*n* = 5–6). **c** Immunohistochemical staining of metadherin (MTDH) protein in tumor tissue in various treatment groups. Abbreviation: *PBS* phosphate-buffered saline

The MCF-7 cells were injected subcutaneously into BALB/c nude mice. When the tumors reached a size of about 100 mm^3^, Cy5.5-labeled NP-TAX–siRNA were injected by the tail vein. In vivo imaging results showed that the NPs gradually accumulated in tumor sites as a function of time (Additional file 1: Figure S7). Ten hours after the administration, we observe maximal fluorescence intensity in tumors, and at 24 h, the NPs were excreted from the mice body except for the tumor tissues, which is one of the desired characteristics of nanomaterials for in vivo application. In contrast, no special fluorescence was detected over time for the Cy5.5-labeled free siRNA treated group. We further performed ex vivo imaging assay. The mice were sacrificed after 10 h of administration, and the tumors and major organs (liver, heart, lung, spleen, and kidney) were collected. As can be seen in Fig. 7a, NP-TAX–siRNA accumulates mainly in the tumor tissues, and there is little accumulation in the liver and kidney and barely any in the heart, lung, and spleen.

Apart from that, we evaluated the antitumor activity of NP-TAX–siRNA using MCF-7 tumor-bearing mice. We randomly divided mice bearing about 100 mm^3^ tumors into seven groups: saline, blank NPs, free TAX, free siRNA, NP-TAX, NP-siRNA, and NP-TAX–siRNA. Mice were treated with different vehicles via the tail vein every two days for a total of 21 days. In the saline and blank NPs groups, the tumors grew fast and the mice were sacrificed two days after the last injection because of having large tumor size (~800 mm^3^) (Fig. 7b). Treatment with free siRNA or NP-siRNA has no significant inhibition effect on tumor growth. Although free TAX and NP-TAX slow down the tumor growth to a certain extent, their inhibitory effects are far lesser than the NP-TAX–siRNA.

To confirm the role of MTDH knockdown in the interference of tumor growth, we sectioned tumors and analyzed the levels of MTDH protein. Figure 7c shows that the MTDH expression level was dramatically suppressed by the NP-siRNA and NP-TAX–siRNA treatments compared with that in the saline group. Nevertheless, the free siRNA does not decrease the MTDH expression in tumor tissues. In the blank NP, free TAX, or NP-TAX–treated group, MTDH expression is similar to that in the saline group. In brief, the results suggest that siRNA is capable of effectively diminishing the MTDH gene expression in vivo only when delivered into the tumors by NPs and thus increasing the TAX antitumor effect.

## Discussion

MTDH was identified as an oncogene that functions in both drug resistance and metastasis [16]. Upregulation of the MTDH gene could promote the proliferation of a variety of tumor cells, such as esophageal cancer, gastric cancer, glioma, and breast cancer [17–20]. Previous study showed that overexpression of MTDH induces estrogen-independent growth of MCF-7 breast cancer cells and mediates tamoxifen resistance [21]. Similarly, in our previous studies, overexpression of MTDH enhances the resistance of MDA-MB-231 cells to doxorubicin [9]. In contrast, downregulation of MTDH could inhibit tumor cell growth, induce apoptosis, and increase the sensitivity of tumor cells to chemotherapeutic drugs. In gastric cancer, studies showed that knockdown of MTDH by siRNA in SGC790 cells could apparently inhibit cell proliferation by blocking cell cycle in G_0_/G_1_ phase [18].

In our study cohort of 44 patients with breast cancer, we found that MTDH expression was negatively correlated with the probability of DFS and efficacy of TAX treatment, which should be further confirmed in a large population of patients with breast cancer. At cellular level study, we tested the cell proliferation of MCF-7–MTDH and MCF-7–MTDH–shRNA cells by CCK-8 assay and found that MTDH knockdown inhibits cell growth. The results of flow cytometry demonstrated that knockdown of MTDH resulted in an increase of G_0_/G_1_ phase cells and reduction of S and G_2_/M phase cells but that MTDH overexpression induced cell cycle arrest in S phase. Additionally, knockdown of MTDH inhibits the growth of xenograft tumor in vivo. Taken together, our results suggest that MTDH expression plays a crucial role in the MCF-7 breast cancer cell proliferation and is potentially useful for breast cancer treatment.

As one of the most important anticancer drugs, TAX has been widely used for chemotherapy in various malignant tumors for about 40 years [22] and is the first-line chemotherapy drug in breast cancer therapy [23]. By stabilizing the microtubule polymer and preventing microtubules from disassembly, TAX arrests the cell cycle in the G_2_/M phase and induces cell apoptosis [23–25]. However, the chemotherapy resistance is a major limitation of its effect and impacts the prognosis of patients with breast cancer. In the present work, we determined the sensitivity of wild type of MCF-7 (MCF-7–MTDH) and MTDH silencing cell (MCF-7–MTDH–shRNA) to TAX treatment. The results suggested that MCF-7–MTDH–shRNA was inhibited by TAX with a much higher rate than MCF-7–MTDH. We further examined the cell apoptosis rate and found that the apoptosis induced by TAX in MCF-7–MTDH–shRNA cells was higher than that in MCF-7–MTDH cells. Furthermore, the percentage of G_2_/M phase in MCF-7–MTDH–shRNA treated with TAX was significantly higher than that in the control and MCF-7–MTDH groups. In in vivo experiments, compared with the MCF-7–MTDH and control groups, the volume of MCF-7–MTDH–shRNA xenograft tumors treated with TAX was significantly smaller. These data together suggest that overexpression of MTDH resisted TAX but that MTDH knockdown increased the sensitivity of MCF-7 cells to TAX treatment. In addition, MTDH plays a key role in the activation of diverse signaling pathways, including PI3K/Akt, NF-κB, and Wnt/β-catenin pathways [3, 26]. The activation of NF-κB is critical to the resistance of tumor cells to cytotoxic agents and microtubule-disrupting agents [27, 28]. We also examined the protein expression level of p65 and IκBα in various MCF-7 cells and showed that MTDH overexpression was correlated with chemoresistance to TAX but that MTDH knockdown increased the sensitivity of TAX by inhibiting the NF-κB/IκBα pathway. This implies that one can increase the effectiveness of TAX treatment on breast cancer by lowering MTDH expression in tumor cells.

Chemoresistance is currently the major cause for breast cancer treatment failure, especially for metastatic breast cancer. Numerous siRNAs have been demonstrated to be effective for in vivo tumor growth modulations [29], but the delivery of siRNAs in vivo has been challenging for antitumor therapy because of their instability in physiological conditions, improper cellular distribution, and low bioactivity [30]. Naked siRNA has a short half-life in the bloodstream because of rapid degradation by nucleases in plasma or excreted by kidney [31]. Moreover, owing to high molecular weight, hydrophilic properties, and high density of charge, naked siRNA hardly penetrates across cell membranes [32]. Using NPs, especially the biodegradable polymer NPs to load siRNA can realize controlled and targeted drug delivery with high efficacy and low side effects [33, 34]. Also, the polymeric NPs readily realize the co-delivery of siRNA with hydrophobic or hydrophilic drugs [35]. In order to reverse drug resistance and improve the utilization of drug effectively, researchers have developed multiple nanocarriers and different dosage forms, such as NP albumin-bound TAX [36]. In this study, for tumor-specific MTDH knockdown, we constructed an amphiphilic PLGA-based copolymer NP for co-delivery of anti-MTDH siRNA and TAX into tumors. In vivo imaging results showed that the two drugs-loaded NPs (NP-TAX–siRNA) accumulated mainly in the tumor tissues, because of the passive targeting ability from the enhanced penetration and retention (EPR) effect of tumor vessels [37, 38], and inhibited tumor growth dramatically, further confirming that MTDH silencing effectively enhances the TAX therapeutic efficiency. In addition, throughout the whole therapeutic experiment, neither weight loss nor tissue damage was observed in the NP-TAX–siRNA–treated mice, indicating the biosafety of NP-TAX–siRNA for tumor treatment.

## Conclusions

In summary, we have revealed, for the first time, that overexpression of MTDH in breast cancer cells is related to TAX chemotherapeutic drug resistance. To achieve in vivo therapeutic assessment by tumor-specific knockdown of MTDH, we have devised a polymer-based nanocarrier to co-deliver anti-MTDH siRNA and TAX into tumor tissues. The designed NPs were composed of a cationic copolymer, which wrapped TAX in the inside and adsorbed the negatively charged siRNA on their surface. After systemic administration, the NPs had good tumor-targeting ability based on the EPR effect of tumor vasculatures and displayed effective antitumor activity without overt side effects. Based on our study, we provide a new strategy for reversing TAX resistance in breast cancer treatment, especially for those with high MTDH protein level.

## Additional file

Additional file 1: **Table S1**. Characteristics of 44 breast cancer patients with neoadjuvant chemotherapy. **Figure S1**. The MTDH mRNA and protein expressions level in different groups after transfecting. We constructed four groups of shRNA and one control shRNA. Then we selected the optimal silent shRNA via real-time PCR and western blot. **Figure S2**. Annexin V-PE/7-AAD assay for determination of apoptosis of cells overexpressing or knocking down metadherin (MTDH) with a flow cytometer. Annexin-positive cells are presented in gate 4. **Figure S3**. Annexin V-PE/7-AAD assay for the determination of apoptosis of different cells after paclitaxel (TAX) treatment. The apoptosis rate of MCF-7–metadherin–short hairpin RNA (MCF-7–MTDH–shRNA) cells was significantly enhanced. **Figure S4**. Flow cytometry was adopted to analyze cell cycle after cells overexpressing or knocking-downing MTDH. Compared to MCF-7 cell, the MCF-7-MTDH cell had more S phase and less G0/G1 and G2/M phases, while knockdown of MTDH did the opposite. **Figure S5**. Cell cycle assay for different cells after paclitaxel (TAX) treatment. Compared with MCF-7 and MCF-7-vector, the G2/M phase rate of MCF-7–metadherin–short hairpin RNA (MCF-7–MTDH–shRNA) cells was significantly enhanced. While overexpression of MTDH did the opposite. **Figure S6**. Paclitaxel (TAX) release from the polymer nanoparticles (NPs). The NPs showed a faster release rate for TAX over time in PBS at pH 4.4 than at pH 7.4. Each bar represents the mean ± standard deviation of three replicates. **Figure S7**. In vivo tumor targeting of nanoparticles (NPs). Nude mice bearing MCF-7 tumors (~100 mm3) were given a single intravenous injection of Cy5.5-labeled free small interfering RNA (siRNA) or NP-TAX–siRNA by the tail vein. In vivo fluorescence signals were recorded by using a Maestro2.10.0 imaging system for up to 24 h post-injection. Abbreviation: TAX paclitaxel. (DOC 2597 kb)

## Abbreviations

CCK-8: Cell Counting Kit-8
DFS: Disease-free survival
EPR: Enhanced penetration and retention
HRP: Horseradish peroxidase
IP: Intraperitoneal
IRS: Immunoreactive score
MTDH: Metadherin
NF-κB: Nuclear factor-kappa B
NP: Nanoparticle
OS: Overall survival
PBS: Phosphate-buffered saline
pCR: Pathologic complete response
PEI: Poly(etherimide)
PLGA: Poly(lactic-co-glycolic acid)
PP: Percentage of positively stained tumor cells
PVA: Poly(vinyl alcohol)
rpm: Revolutions per minute
RT-PCR: Real-time polymerase chain reaction
SI: Staining intensity
siRNA: Small interfering RNA
TAX: Paclitaxel
