# Supplementary material for: Efficient and tumor-specific knockdown of MTDH gene attenuates paclitaxel resistance of breast cancer cells both in vivo and in vitro
Source: Breast Cancer Res. 2018 Sep 18;20:113. doi: 10.1186/s13058-018-1042-7 (PMC6145322; doi:10.1186/s13058-018-1042-7)
Supplement: Supplementary file 1 — Table S1. Characteristics of 44 breast cancer patients with neoadjuvant chemotherapy. Figure S1. The MTDH mRNA and protein expressions level in different groups after transfecting. We constructed four groups of shRNA and one control shRNA. Then we selected the optimal silent shRNA via real-time PCR and western blot. Figure S2. Annexin V-PE/7-AAD assay for determination of apoptosis of cells overexpressing or knocking down metadherin (MTDH) with a flow cytometer. Annexin-positive cells are presented in gate 4. Figure S3. Annexin V-PE/7-AAD assay for the determination of apoptosis of different cells after paclitaxel (TAX) treatment. The apoptosis rate of MCF-7–metadherin–short hairpin RNA (MCF-7–MTDH–shRNA) cells was significantly enhanced. Figure S4. Flow cytometry was adopted to analyze cell cycle after cells overexpressing or knocking-downing MTDH. Compared to MCF-7 cell, the MCF-7-MTDH cell had more S phase and less G0/G1 and G2/M phases, while knockdown of MTDH did the opposite. Figure S5. Cell cycle assay for different cells after paclitaxel (TAX) treatment. Compared with MCF-7 and MCF-7-vector, the G2/M phase rate of MCF-7–metadherin–short hairpin RNA (MCF-7–MTDH–shRNA) cells was significantly enhanced. While overexpression of MTDH did the opposite. Figure S6. Paclitaxel (TAX) release from the polymer nanoparticles (NPs). The NPs showed a faster release rate for TAX over time in PBS at pH 4.4 than at pH 7.4. Each bar represents the mean ± standard deviation of three replicates. Figure S7. In vivo tumor targeting of nanoparticles (NPs). Nude mice bearing MCF-7 tumors (~100 mm3) were given a single intravenous injection of Cy5.5-labeled free small interfering RNA (siRNA) or NP-TAX–siRNA by the tail vein. In vivo fluorescence signals were recorded by using a Maestro2.10.0 imaging system for up to 24 h post-injection. Abbreviation: TAX paclitaxel. (DOC 2597 kb) [file 13058_2018_1042_MOESM1_ESM.doc]

**Supplementary Information**

**Efficient and tumor-specific knockdown of MTDH gene attenuates paclitaxel resistance of breast cancer cells both in vivo and in vitro**

Li Yang^1#^, Yanhua Tian^2#^, Wei Sun Leong^3#^, Heng Song^4^, Wei Yang^1^, Meiqi Wang^1^, Xinle Wang^1^, Jing Kong^3^, Baoen Shan^1^, Zhengchuan Song^1^*

**Table S1. Baseline Characteristics of the Patients.**

| **Characteristic** | **MTDH low**  **(n=15)** | **MTDH high**  **(n=29)** | **P value** |
| --- | --- | --- | --- |
|  | **No. (%)** | **No. (%)** |  |
| Age |  |  | 0.759 |
| <50 | 9 (60.0) | 16 (55.2) |  |
| ≥50 | 6 (40.0) | 13 (44.8) |  |
| Tumor size (cm) |  |  | 0.455 |
| ≤5 | 11 (73.3) | 18 (62.1) |  |
| >5 | 4 (26.7) | 11 (37.9) |  |
| Stage |  |  | 0.759 |
| ⅡB-ⅢA | 6 (40.0) | 13 (44,8) |  |
| ⅢB-ⅢC | 9 (60.0) | 16 (55.2) |  |
| pCR | 5(33.3) | 5(17.2) |  |
| ER status |  |  | 0.919 |
| positive | 8 (53.3) | 15 (51.7) |  |
| negative | 7 (46.7) | 14 (48.3) |  |
| PR status |  |  |  |
| positive | 6 (40.0) | 16 (55.2) | 0.759 |
| negative | 9 (60.0) | 13 (44.8) |  |
| HER2 status |  |  |  |
| positive | 2 (13.3) | 5 (17.2) | 0.937 |
| negative | 10 (66.7) | 18 (62.1) |  |
| unknown | 3 (20.0) | 6 (20.7) |  |
| Ki-67 |  |  |  |
| ≤20% | 4(26.7%) | 4(13.8%) | 0.576 |
| ＞20% ≤50% | 7(46.7%) | 16(55.2%) |  |
| ＞50% | 4(26.7%) | 9(31.0%) |  |

pCR = pathologic complete response (Can`t find malignant tumor tissues in the breast primary lesions, or only carcinoma in situ in the remaining ingredients). ER=estrogen receptor. PR=progesterone receptor. HER2=human epidermal growth factor receptor 2.


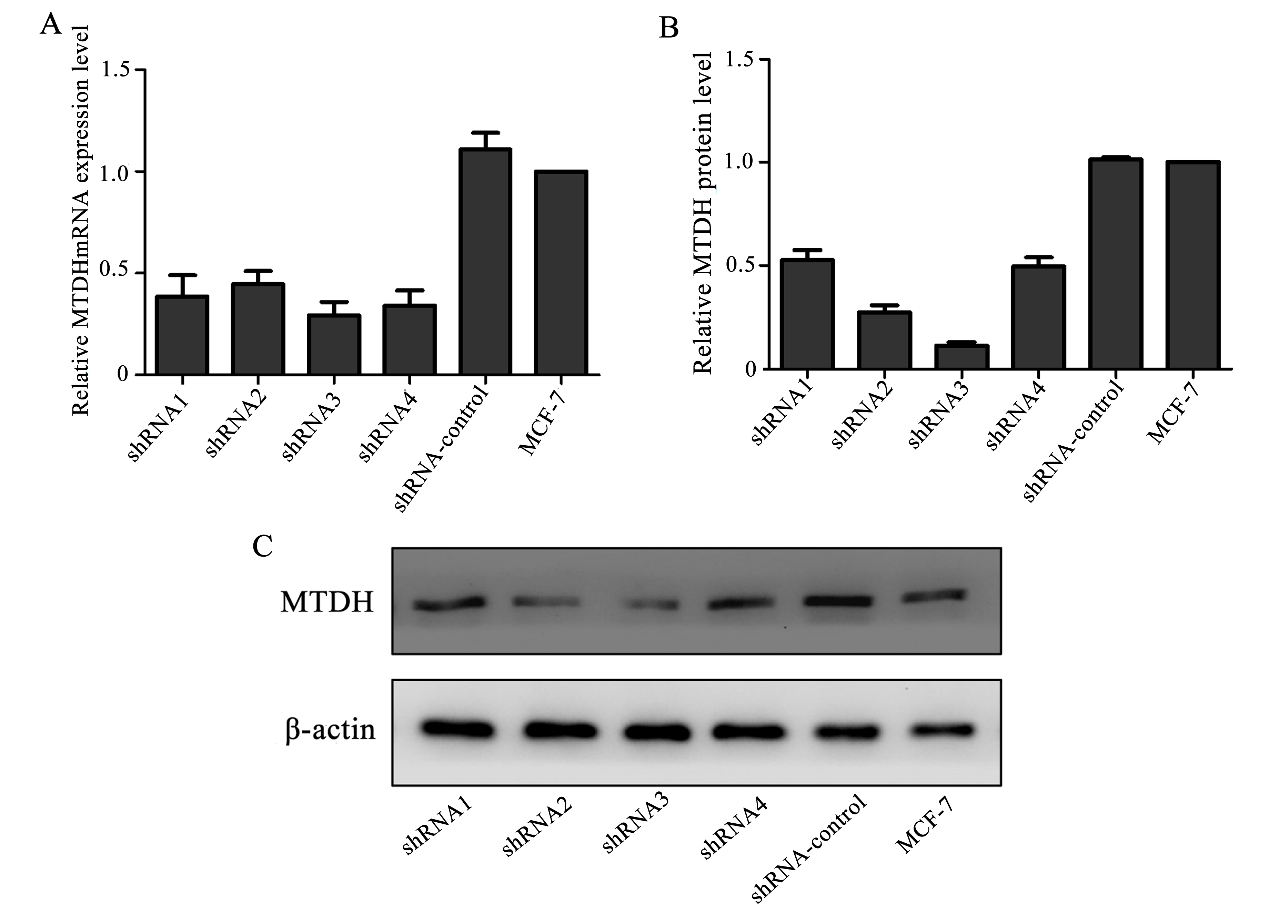


**Figure S1.** The MTDH mRNA and protein expressions level in different groups after transfecting. We selected the optimal silent shRNA (MTDH-shRNA3: 5'-GCAATTGGGTAGACGAAGAAA-3') via real-time PCR and western blot. **A** The MTDH mRNA expressions level of MTDH-shRNA1，MTDH-shRNA2，MTDH-shRNA3，MTDH-shRNA4 and shRNA-control. **B and C** The protein expressions level of MTDH-shRNA1, MTDH-shRNA2, MTDH-shRNA3, MTDH-shRNA4 and shRNA-control.


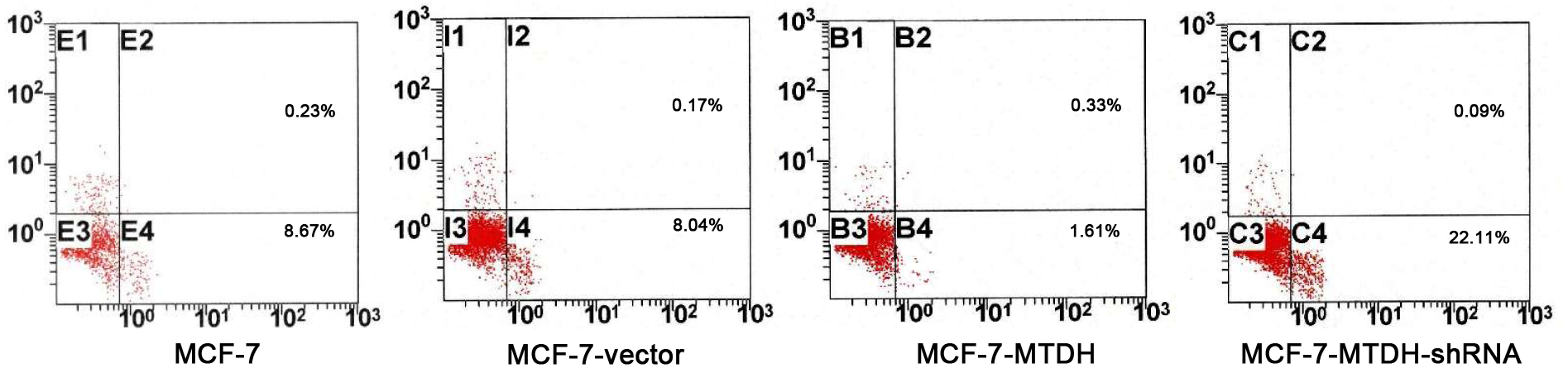


**Figure S2.** Annexin V-PE/7-AAD assay for determination of apoptosis of cells overexpressing or knocking-downing MTDH with a flow cytometer. MCF-7 and MCF-7-vector were used as control. Annexin-positive cells were presented in Gate 4.


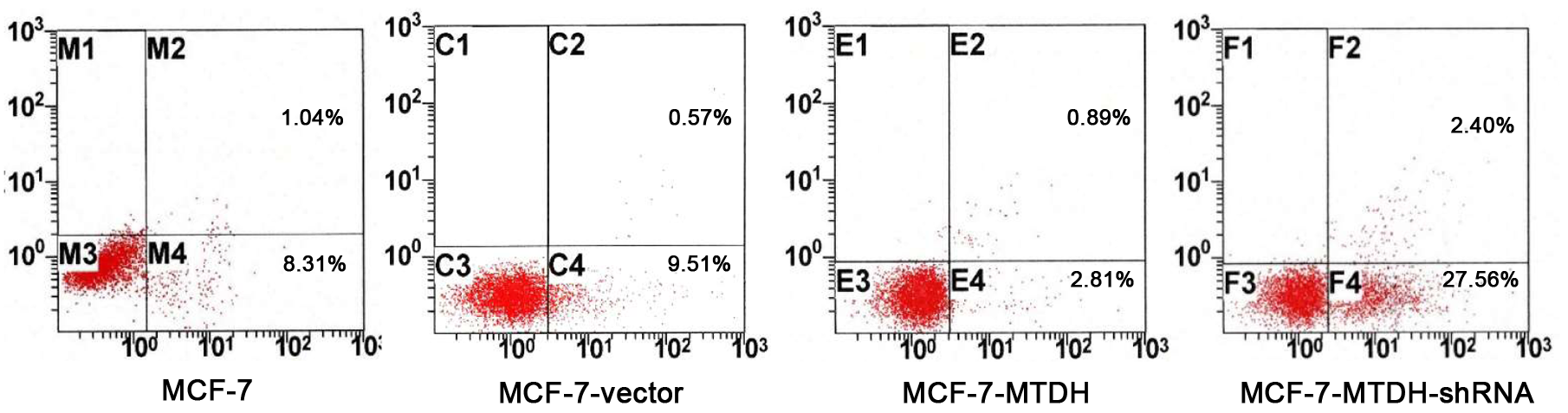


**Figure S3.** Annexin V-PE/7-AAD assay for determination of apoptosis of different cells after TAX treatment. The apoptosis rate of MCF-7-MTDH-shRNA cells was significantly enhanced as shown by the presence of Annexin-positive cells in Gate 4.


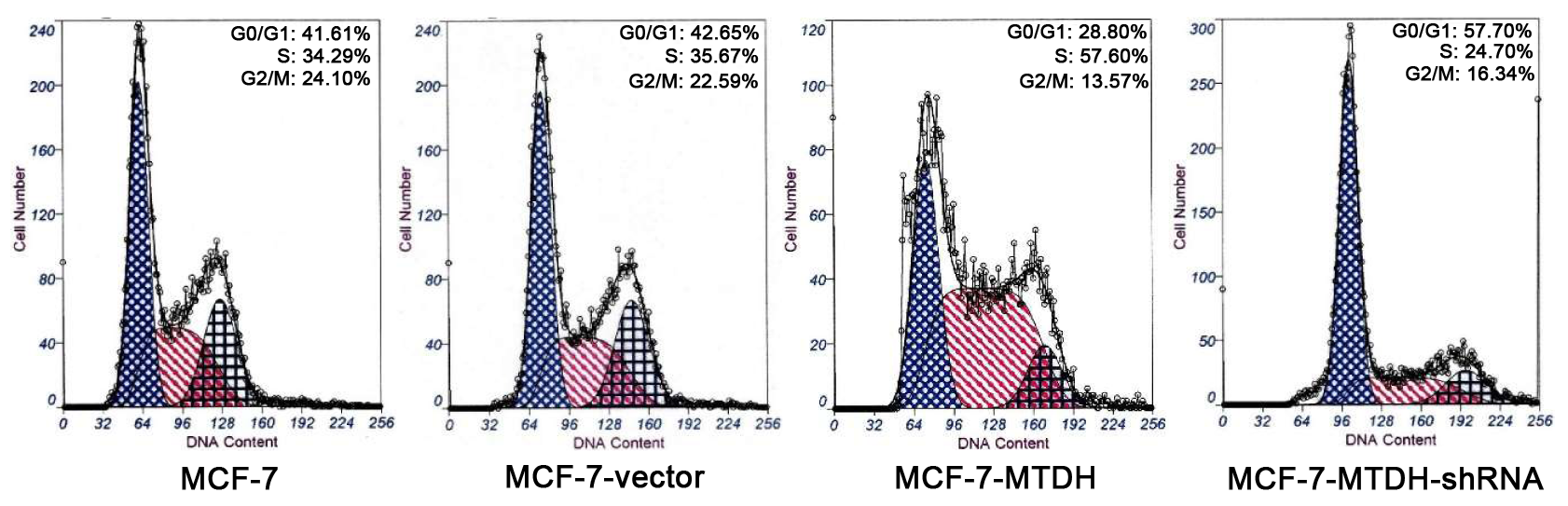


**Figure S4.** Flow cytometry was adopted to analyze cell cycle after cells overexpressing or knocking-downing MTDH. Overexpression of MTDH arrested cells in S phase, while MTDH silencing increased the proportion of G0/G1 phase.


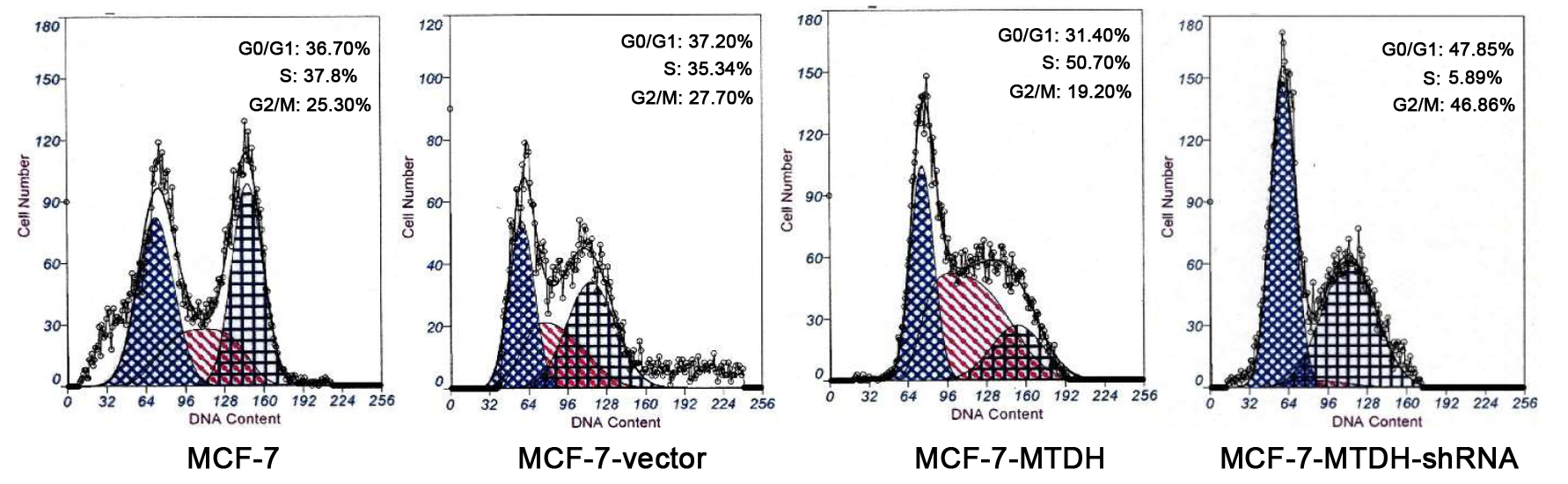


**Figure S5.** Cell-cycle assay for different cells after TAX treatment. The G2/M phase rate of MCF-7-MTDH-shRNA cells was significantly enhanced as shown by the presence of G2/M cells in Gate 4.

**Figure S6.** TAX release from the polymer NPs. Th-NPs were resuspended in PBS at either pH 7.4 or 4.4, and transferred to dialysis bags placed in 30 ml of PBS with stirring at 110 rpm/37 °C. At the indicated time points, the environmental buffer solution was removed, and detected for TAX amount by UV-absorbance assay. The NPs showed a faster release rate for TAX over time in PBS at pH 4.4 than at pH 7.4. Each bar represents the mean ± SD of three replicates.


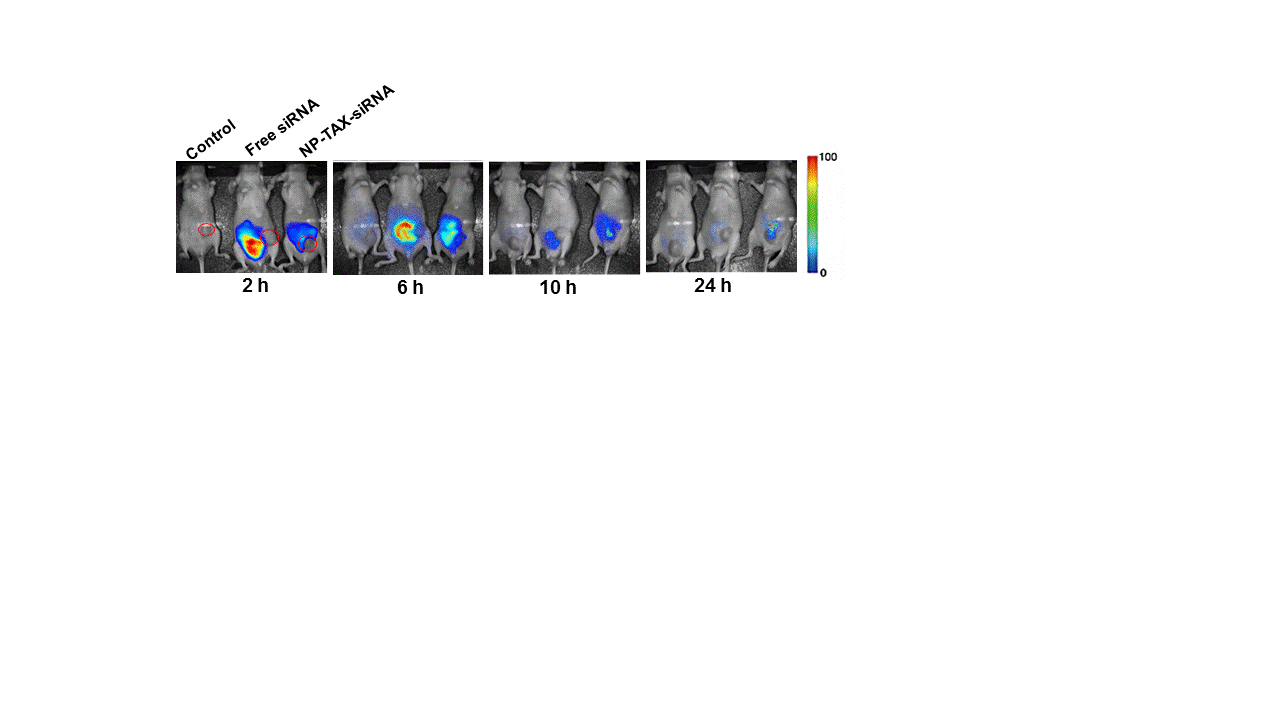


**Figure S7. *In vivo* tumor targeting of the NPs.** Nude mice bearing MCF-7 tumors (~100 mm^3^) were given a single intravenous injection of Cy5.5-labeled free siRNA or NP-TAX-siRNA by tail vein. *In vivo* fluorescence signals were recorded using a Maestro 2.10.0 imaging system for up to 24 hours post-injection. The red circles indicate the tumor.
